# Prevalence and associated factors of tuberculosis among isoniazid users and non-users of HIV patients in Dessie, Ethiopia

**DOI:** 10.1038/s41598-022-16437-3

**Published:** 2022-08-05

**Authors:** Wondwosen Mebratu, Shambel Wedajo, Semira Mohammed, Abel Endawkie, Yeshiwork Damtew

**Affiliations:** 1 Department of Epidemiology and Biostatistics, School of Public Health, College of Medicine and Health Science, Wollo University, Dessie, Ethiopia; 2 Department of General Master of Public Health, School of Public Health, College of Medicine and Health Science, Wollo University, Dessie, Ethiopia; 3 Department of Masters of Business Administration, College of Business and Economics, Wollo University, Dessie, Ethiopia

**Keywords:** Diseases, Medical research

## Abstract

Tuberculosis (TB) is major public health concern and Isoniazid Preventive Therapy (IPT) helps to prevent TB development among patients living with human immune deficiency virus (PLWHIV). However, the evidence is limited especially in the study area. Therefore, this study aimed to determine the prevalence and factors associated with TB among IPT users and non-IPT users of PLWHIV in Dessie, Ethiopia. A comparative cross-sectional study was employed for1 month in Dessie. A total of 326 respondents were selected using systematic random sampling. Bivariable and multivariable logistic regression analyses were computed to identify factors associated with Tuberculosis. In multivariable analysis, AOR with 95% CI was used to declare statistically significant variables with TB. The prevalence of TB among non-IPT users was 48.5%, (95% CI 40.8–56.2%), and among IPT users was 8%, (95% CI 5–13%). Cotrimoxazole prophylaxis therapy (CPT) (AOR = 5.835, 95% CI 2.565–13.274), IPT (AOR = 10.359, 95% CI 4.054–26.472), ART adherence (AOR = 30.542, 95% CI 12.871–72.475), and believing that IPT use prevents TB (AOR = 0.093, 95% CI 0.018–0.484) were statistically significant factors. The prevalence of TB was higher among non-IPT users than among IPT users. Therefore, efforts should be strengthened to implement widespread use of IPT among adult PLWHIV.

## Introduction

The initial infection with Mycobacterium Tuberculosis in healthy individuals is usually asymptomatic as the immune system strives to "wall off" the germs, resulting in a latent TB infection^1–3^. Active tuberculosis can be diagnosed via direct microscopy (sputum smear analysis), mycobacterium culture, chest X-ray, pathologic investigation, and/or clinical features. Active tuberculosis is effectively treated with a six-month course of medicines^3–5^.

Tuberuclosis is the greatest cause of mortality among HIV-positive people worldwide, and HIV is the most powerful risk factor for active tuberculosis development^6^. According to a global epidemiological TB data prevalence analysis, about 10 million people had incident TB in 2018 and 1.5 million people died from TB-related causes, representing 2% and 5% decreases over 2017^6,7^. However, in 2018, 70 percent of the estimated 10 million people with TB were reported to WHO as having HIV co-infection, up 9.4% from 2017^6–8^. Globally, the prevalence of TB/HIV co-infection is high, with 90% of cases occurring in developing nations^9,10^. Sub-Saharan Africa is responsible for 79 percent of the global TB/HIV co-infection burden^6,7^. With an estimated incidence of 579 per 100,000 for all varieties of TB and accounting for 7% of all fatalities in the country, Ethiopia is ranked seventh among the 22 countries with high TB burdens^11–13^.

Antiretroviral therapy (ART) was used in over 57 percent of HIV-positive TB patients in Africa, with 520,000 HIV-positive people receiving TB preventive therapy (TPT)^14^. In 2016, an estimated 16,000 (PLWHIV) Ethiopians developed tuberculosis, with 8,625 (54%) were on co-treatment^9^. Due to the HIV pandemic, Sub-Saharan Africa, especially Ethiopia, has the highest socio-economic burden of TB/HIV co-infection, which disproportionately affects the most economically active age groups^5,14^. Active TB prevention is a critical technique for reducing socio-economic burdens^14,15^. Isoniazid Preventive Therapy (IPT) was administered to all HIV positive patients once a health care provider ruled out an active TB. WHO recommended that IPT is one of the components of PLHIV's comprehensive treatment, and it is highly advisable to be started and continued at the HIV care/ART clinic for PLWHIV who were found to be negative for active TB. For HIV positive clients without active TB who receive IPT, the patient's details are recorded in the ART and Pre-ART registration^16^.

The WHO recommended a 36-month course of IPT to prevent TB infection among HIV-positive people living in high-TB-endemic areas^14,17^. Ethiopia's Federal Ministry of Health recently created and developed national guidelines on tuberculosis prevention and control, which included in a health sector transformation plan based on policy guidance and global and national epidemiologic conditions^18^. PLWHIV with a positive tuberculin skin test (TST) appears to be a strong predictor for the potential value of IPT in both high- and low-burden TB settings, according to some studies^19^, reinforcing the WHO recommendation that TST should guide testing wherever practicable^19,20^. Without TST, IPT increases the risk of inadvertent monotherapy treatment of active TB, as well as the development of isoniazid and polydrug resistance^21^.

Despite the fact that there is solid evidence for the use of IPT and a global recommendation that it is to be used as part of routine care for all HIV-infected persons after ruling out active TB. The coverage and implementation of IPT have been delayed in many countries, including Ethiopia^14^. These difficulties were linked to the development of isoniazid-related resistance, drug side effects, clinicians' perceptions toward its effectiveness, failure to use TB screening algorithms on a regular basis, and drug supply limitations^22,23^. TST is not used to initiate IPT in Ethiopia, but IPT is initiated based on symptomatic screening to rule out the presence of active tuberculosis; nevertheless, this may limit IPT effectiveness by missing patients with latent tuberculosis^9,10,14^. Furthermore, individuals may get tuberculosis (TB) disease after finishing IPT^24^. Because the benefits of prolonged therapy differed by demographic factors, multiple studies have suggested that further researches are to be done to determine whether IPT is useful in the local HIV population and in all populations in the world^11,12,25,26^. According to various studies, socio-demographic factors (age, educational status, occupation, and place of residence), knowledge of TB and IPT, behavioral factors (smoking, alcohol consumption), clinical and laboratory characteristics (past opportunistic infection (OI), WHO clinical stage, OI treatment other than INH, and weight) were all linked to TB among PLWHIV^11,12,25–27^.

However, there is inadequate evidence about the prevalence of TB and associated factors among IPT and non- IPT users of PLWHIV in Ethiopia, particularly in the Amhara region, Dessie City administration. The findings of this study may help to improve the planning and service delivery of health professionals, zonal and regional health managers, program planers and other stakeholders in the study area and it helps to call for programmer to revise current strategies by ensuring that the various factors contributing to non-IPT and IPT users on TB prevalence among PLWHIV. Infact, IPT among PLWHIV had considerable implications for efforts to achieve SDG targets by 2030 to end the epidemics of communicable diseases of specially TB; further intention to identify factors that favor the development of TB among PLWHIV may be useful to identify areas of future focus in the control and management of TB among non-users of IPT and users of IPT of PLWHIV. Therefore, the aim of this study is to determine the prevalence and factors associated with TB among IPT and non-IPT users of PLWHIV in Dessie, Northeast, Amhara, Ethiopia.

## Results

### Socio-demographic characters of respondents

In this study, 163 IPT users and 163 non-IPT users of PLHIV were included. The overall response rate was 100%. The mean age of the patients with standard division is 38.83 ± 11.74 respectively. Among IPT users 61 (37.4%) and among non-IPT users 51 (31.3%) were in the age group between 31 and 40 years. With respect to sex, the female IPT users and non-IPT users was 93 (57.1%), similar proportion to each group. Approximately, IPT users (77.3%) and non-IPT users (79.1%) were urban residents. With educational background, among IPT users and non-IPT users having secondary and above level of education were 65 (39.9%) and 71 (43.6%), respectively. Regarding marital status, IPT users (44.8%) and non-IPT users (42.3%) were married, followed by 69 (42.3%) and 60 (36.8%) were windowed/divorced and, respectively (Table 1).

**Table 1:** Socio-demographic characteristics of respondents by category of PLHIV Dessie city administrative 2021.

Characteristics’	IPT user	Non IPT user	Total
**Age**			
18–30 years	40 (24.5%)	52 (31.9%)	92 (28.2%)
31–40 years	61 (37.4%)	51 (31.3%)	112 (34.4%)
41–50 years	36 (22.1%)	36 (22.1%)	72 (22.1%)
> 50 years	26 (16.0%)	24 (14.7%)	50 (15.3%)
**Sex**			
Male	70 (42.9%)	70 (42.9%)	140 (42.9%)
Female	93 (57.1%)	93 (57.1%)	186 (57.1%)
**Occupation**			
House wife	33 (20.2%)	42 (25.8%)	75 (23.0%)
Merchant	22 (13.5%)	27 (16.6%)	49 (15.0%)
Daily labor	52 (31.9%)	38 (23.3%)	90 (27.6%)
Gov’tal employee	30 (18.4%)	31 (19.0%)	61 (18.7%)
Others^1^	26 (15.9%)	25 (15.6%)	51 (12.1%)
**Level of education**			
Illiterate	55 (33.7%)	47 (28.8%)	102 (31.3%)
Read and write	7 (4.3%)	6 (3.7%)	13 (4.0%)
Primary (1–8)	36 (22.1%)	39 (23.9%)	73 (23.0%)
Secondary and above	65 (39.9%)	71 (43.6%)	136 (41.7%)
**Marital status**			
Single	21 (12.9%)	34 (20.9%)	55 (16.9%)
Married	73 (44.8%)	69 (42.3%)	142 (43.6%)
Windowed/divorced	69 (42.3%)	60 (36.8%)	129 (39.6%)
**Family size**			
1–3	119 (73.0%)	119 (73.0%)	238 (73.0%)
> 3	44 (27.0%)	44 (27.0%)	88 (27.0%)

### Clinical profiles of respondents

In this study, among the study subjects, 127 (77.9%) of IPT users and 104 (63.8%) of non-IPT users were found at normal BMI classification (18.5 to 25 kg/m^2^). One hundred fifty-five IPT users (95.1%) and 124 (76.1%) non-IPT users were found to be working functionally. One hundred forty-two IPT users (87.1%) and 101 (62.0%) non-IPT users were on the stage one WHO clinical staging systems. Thirty-six percent of IPT users and 63.0% of non-IPT users had ever experienced opportunistic infection. Among the respondents, 48 (29.4%) of IPT users and 77 (47.2%) of non-IPT users had CD4 counts less than 350. Additionally, seventeen (10.4%) of IPT users and 67 (41.1%) of non-IPT users had poor adherence to ART.

In this study, the occurrence of TB among IPT users and non-IPT users were 8% and 48.5%, respectively (Table 2).

**Table 2:** Clinical-related characteristics of PLWHIV in Dessie city administrative 2021.

Characteristics	IPT user	Non IPT user	Total
**BMI**			
Below 18.5	10 (6.1%)	48 (29.4%)	58 (14.3%)
18.5–25	127 (77.9%)	104 (63.8%)	231 (70.9%)
Greater than 25	26 (16.0%)	11 (6.7%	37 (11.3%)
**Current functional status**			
Working	155 (95.1%)	124 (76.1%)	279 (85.6%)
Ambulatory and bed redden	8 (4.9%)	39 (23.9%)	47 (14.4%)
**Current WHO stage**			
Stage 1	142 (87.1%)	101 (62.0%)	243 (74.5%)
Stage 2	13 (8.0%)	11 (6.7%)	24 (7.4%)
Stage 3	5 (3.1%)	39 (23.9%)	44 (13.5%)
Stage 4	3 (1.8%)	12 (7.4%)	15 (4.6%)
**Ever experienced OI**			
Yes	59 (36.2%)	103 (63.0%)	162 (49.5%)
No	104 (63.8%)	60 (37.0%)	164 (50.5%)
**Current CD4 count**			
Greater than 350	115 (70.6%)	86 (52.8%)	201 (61.7%)
Less than 350	48 (29.4)	77 (47.2%)	125 (38.3%)
**Adherence to ART**			
Good	146 (89.6%)	96 (58.9%)	242 (74.2%)
Poor	17 (10.4%)	67 (41.1%)	84 (25.8%)
**Occurrence of TB**			
Yes	13 (8.0%)	79 (48.5%)	92 (28.2%)
No	150 (92.0%)	84 (51.5%)	234 (71.8%)
**CPT**			
Yes	116 (71.2%)	86 (52.8%)	202 (62.0%)
No	47 (28.8%)	77 (47.2%)	124 (38.0%)

### Knowledge toward isoniazid preventive therapy and behavior-related characteristics

Approximately 61.3% of IPT users and 57.1% of non-IPT users had ever heard any information about isoniazid preventive therapy. Among those who had ever heard any information about IPT, the source of information for 93.0% of IPT users and 91.4% of non-IPT users were health care workers. Most IPT users (96.3%) and non-IPT users (91.4%) believe that isoniazid preventive therapy prevents tuberculosis. Regarding the behavioral practice of respondents, among IPT users and non-IPT users who had ever smoked cigarettes were 8% and 9.8%, respectively. Among those ever smoking, 84.6% of IPT users and 81.3% of non-IPT users had information that smoking is not advised for HIV clients. Approximately 16.6% of IPT users and 16% of non-IPT users had ever drunk alcohol. Most IPT users (70.6%) and 69.8% of non-IPT users disclosed their status to relatives/family (Table 3).

**Table 3:** Knowledge and attitude on isoniazid preventive therapy and behavior-related characteristics of PLWHIV in Dessie city administrative 2021.

Characteristics	IPT user	Non IPT user	Total
**Ever heard about IPT**			
Yes	100 (61.3%)	93 (57.1%)	193 (59.2%)
No	63 (38.7%)	70 (42.9%)	133 (40.8%)
**Source of information**			
Mass media	3 (3%)	0	3 (1.5%)
Health workers	93 (93.0%)	85 (91.4%)	178 (92.2%)
Family, friends and neighbors	4 (4.0%)	8 (8.6%)	12 (6.3%)
**Think IPT to prevent tuberculosis**			
Yes	157 (96.3%)	149 (91.4%)	306 (93.9%)
No	6 (3.7%)	14 (8.6%)	20 (6.1%)
**Ever smoke**			
Yes	13 (8%	16 (9.8%)	29 (8.9%)
No	150 (92%)	147 (90.2%)	297 (91.1%)
**Ever drinking alcohol**			
Yes	27 (16.6%)	26 (16.0%)	53 (16.3%)
No	136 (83.4%)	137 (84.0%)	273 (83.7%)
**Disclose the status to relatives/family**			
Yes	115 (70.6%)	114 (69.9%)	229 (70.2%)
No	48 (29.4%)	49 (30.1%)	97 (29.8%)

### Prevalence of TB among HIV patients in Both IPT users and non-users of IPT

The prevalence of TB among non-IPT users of PLWHIV was 48.5% (95% CI 40.8–56.2%), he prevalence of TB among IPT users of PLWHIV was 8% (95% CI 5–13%). but.

### Factors associated with TB among non-IPT users of HIV patients

In this study, good ART adherence (AOR = 0.039, 95% CI 0.008–0.178) and ever experiencing opportunistic infection (AOR = 0.007, 95% CI 0.000–0.103) were factors associated with TB among non-IPT users (Table 4).

**Table 4:** Bivariable and multivariable logistic analysis of factors associated with TB among PLWHIV nonusers of isoniazid preventive therapy for TB in Dessie city administrative 2021.

	TB		Total	COR	AOR
	Yes	No			
**Residence**					
Rural	22	12	34	2.316 (1.057–5.075)	1.882 (0.376–9.424)
Urban	57	72	129	1	1
**ARTadherence**					
Good	19	77	96	0.029 (0.011–0.073)	**0.039 (0.008–0.178)**
Poor	60	7	67	1	1
**Disclosure their status**					
Yes	61	53	114	1.982 (0.997–3.942)	3.047 (0.649–14.305)
No	18	31	49	1	1
**CPT**					
Yes	22	64	86	1	1
No	57	20	77	8.291 (4.105–16.745)	2.597 (0.258–26.108)
**CD4 count**					
> 350	22	64	86	0.121 (0.060–0.244)	0.267 (0.024–2.959)
< 350	57	20	77	1	1
**BMI**					
< 18.5	31	17	48	4.863 (1.137–20.788)	0.106 (0.008–1.443)
18.5–25	45	59	104	2.034 (0.510–8.104)	0.192 (0.020–1.874)
> 25	3	8	11	1	1

Significant values are in bold.

### Factors associated with TB among IPT users of HIV patients

In this study, good ART adherence (AOR = 0.030, 95% CI 0.005–0.172) was a factor associated with TB among Isoniazid preventive therapy users (Table 5).

**Table 5:** Bivariable and Multivariable logistic analysis on factors associated with TB among TB preventive therapy users in PLWHIV Dessie city administrative 2021.

	TB		COR	AOR
	Yes	No		
**ART adherence**				
Good	3	143	0.015 (0.003–0.066)	**0.030 (0.005–0.172)**
Poor	10	7	1	1
**CPT**				
Yes	2	114	1	1
No	11	36	17.417 (3.687–82.266)	3.072 (0.036–259.447)
**CD4 count**				
≥ 350	2	113	0.060 (0.013–0.281)	0.389 (0.005–32.300)
< 350	11	37	1	1
**Experience OI**				
Yes	11	48	11.688 (2.493–54.802)	1.666 (0.219–12.668)
No	2	102	1	1

Significant values are in bold.

### Factors associated with TB among non-IPT and IPT users of HIV patients

In bivariable logistic regression analysis, variables such as sex, CD4 count, ART adherence, IPT use, and BMI less than 18.5 kg/m^2^, believing that IPT use prevents TB, ever smoke, -ever drunk alcoholic beverages, and CPT were significantly associated with the outcome variable, Tuberculosis.

### Multivariable logistic regression analysis

During bivariable logistic regression analysis, variables with P-value < 0.25 were entered into multivariable logistic regression, Therefore, factors significantly associated with tuberculosis were Cotrimoxazole prophylaxis therapy (CPT) (AOR = 5.835, 95% CI 2.565–13.274), IPT (AOR = 10.359, 95% CI 4.054–26.472), ART adherence (AOR = 30.542, 95% CI 12.871–72.475), and believing that IPT use prevents TB (AOR = 0.093, 95% CI 0.018–0.484).

After controlling for other variables remains constant, the odds of developing TB among PLWHIV who have used IPT were ten times higher than among those not used IPT [(AOR = 10.359, 95% CI (4.054–26.472)]. The odds of TB among PLWHIV who had poor adherence to ART was thirty times more likely than that of PLWHIV who had good adherence to ART [(AOR = 30.542, 95% CI (12.871–72.475))]. The chance of developing TB among PLWHIV who believe that IPT use prevents TB is 91% less likely than those who believe it does not [(AOR = 0.093, 95% CI (0.018–0.484))]. The odds of developing TB among PLWHIV who were not taking cotrimoxazole prophylaxis was 5.85 [(AOR = 5.835, 95% CI (2.565–13.274)] more likely than those who were taking cotrimoxazole prophylaxis (Table 6).

**Table 6:** Bivariable and multivariable logistic regression analysis of factors associated with TB among isoniazid and non-users IPT of PLWHIV Dessie city administrative 2021.

Variables	Category	TB		COR (95% CI)	AOR (95% CI)
		Yes	No		
Sex	Male	48	92	1.68 (1.036–2.737)	1.660 (0.683–4.032)
	Female	44	142	1	1
Current CD4 count	> 350 cells/μl	24	177	0.114 (0.065–0.198)	1.432 (0.217–9.443)
	< 350 cells/μl	68	57	1	1
ART Adherence	Good	22	220	1	**1**
	Poor	70	14	50.00 (24.3–102.9)	**30.542 (12.871–72.475)**
IPT vs non IPT	IPT user	13	150	1	**1**
	Non IPT user	79	84	10.852 (5.7–20.7)	**10.359 (4.054–26.472)**
Believe IPT use prevent TB	Yes	78	228	0.147 (0.054–0.395)	**0.093 (0.018–0.484)**
	No	14	6	1	1
Ever smoke	Yes	16	13	3.579 (1.6467.783)	3.284 (0.914–11.797)
	No	76	221	1	
Ever drinking alcohol	Yes	21	32	1.867 (1.0113.447)	1.213 (0.340–4.322)
	No	71	202	1	1
BMI	< 18.5 kg/m^2^	35	23	8.52 (2.87–25.278)	0.625 (0.129–3.026)
	18.5–25 kg/m^2^	52	183	1.59 (0.59–4.33)	0.424 (0.111–1.613)
	> 25 kg/m^2^	5	28	1	1
CPT	Yes	24	178	1	**1**
	No	68	56	9.00 (5.176–5.670)	**5.835 (2.565–13.274)**

Significant values are in bold.

## Discussion

The aim of this study was to determine the prevalence of TB and its associated factors among PLWHIV in both IPT users and non-IPT users. The prevalence of TB among non-IPT users was higher than that of IPT users among PLWHIV. This is similar with a study finding in different areas of Ethiopia, which showed that the prevalence of TB was higher among patients with no IPT prophylaxis than among patients with IPT prophylaxis^14,16,28–30,30–32^ and other countries, such as a study performed in Zimbabwe^33^. The possible explanation might be due to IPT prevents the transition of latent infection to active TB disease, may account for the difference in tuberculosis development among HIV patients. As a result, providing IPT may help to boost TB preventive and control methods among adult HIV-positive patients.

ART adherence was a significant impact in the development of TB. In this study, the occurrence of TB among PLWHIV with poor adherence to ART was thirty times higher than that of people with good ART adherence. This is consistent with a study performed in Jimma Ethiopia and Southeast Nigeria^28,29,34^. The possible reason might be due to that poor ART adherence has contributed to increasing viral proliferation among HIV patients. As result, the link between TB and poor adherence to ART is most likely owing to the loss of ART's immunity-boosting impact when viral proliferation increased^18^. Poor adherence can cause ART to fail to adequately suppress the virus, this may weaken the immune systems of HIV patients, thereby exposing patients to tuberculosis.

Believing that IPT use prevents TB was found to be significantly associated with TB among PLWHIV. In this study, the chance of developing TB among PLWHIV who believe that IPT use prevents TB was 91% than those who believe that it does not prevent it^29^. This may be due to good attitudes displayed by participants that could strengthen IPT uptake, which decreases the risk of TB. Unfavorable attitudes could create challenges for TB prevention practice intentions and behavior toward IPT.

Cotrimoxazole prophylaxis was found to be a preventive factor against tuberculosis among PLWHIV. This is supported by research findings from Asia, South Africa, and Switzerland^10,35,36^. This is probably because of that Cotrimoxazole has a wide range of antibacterial, antifungal, and antiparasitic properties. Specifically, sulfamethoxazole is found to be active against Micobacterium TB in vitro in several drug trials^10^. Several observational studies found that cotimoxazole reduced new TB incidence in HIV-infected people, implying that cotrimoxazole has a direct antitubercular effect^36,37^. Furthermore, cotrimoxazole prophylaxis has beneficial effects in enhancing CD4 count and reducing viral load^37^.

## Conclusions

There was a significant difference in the prevalence of tuberculosis between non-isoniazid preventive therapy users and isoniazid preventive therapy users among patients living with HIV. As a result, the prevalence of tuberculosis among patients living with HIV was higher for non-isoniazid preventive therapy users than among isoniazid preventive therapy users. The use of isoniazid preventive therapy and cotrimoxazole preventive therapy, ART adherence, and belief that isoniazid preventive therapy use prevents TB were independent factors affecting the development of TB among HIV patients. Therefore, efforts should be strengthened to implement widespread use of ART, CPT, IPT among adult PLWHIV and creating awareness among HIV patients about the importance of IPT for the prevention of TB. Finally, we recommend for researchers to conduct prospective cohort studies and randomized controlled trials to identify the real and timely impact of ART, CPT and IPT for TB development among PLWHIV independently (Supplementary Information S1).

## Methods and materials

### Study area

This study was conducted at health facilities of Dessie City administration. Dessie is one of the Amhara regional states of the metropolitan zone. It is located 401 km away from Addis Ababa, the capital city of Ethiopia, and 486 km from Bahir-Dar, the regional town and serving more than 223,439 people at the end of the year 2019. Administratively, Dessie city is divided into five administrative sub-cities. In 2019, there was a total of one comprehensive specialized hospital, one primary hospital, 8 public health centers, 6 health posts, 3 private hospitals, 9 private primary clinics, 27 medium clinics, 23 drug stores, 24 pharmacies and 3 non-governmental health facilities that provide health services in Town. This study was employed where ART, TB screening, IPT Prophylaxis, and OI treatment services were provided, particularly in 2 hospitals, 3 public health centers, and 4 private health facilities.

### Study design, period, and population

A facility-based comparative cross-sectional study was conducted from February 1/2020 to March 1/2020. All adult PLHIV enrolled ART care follow-up with IPT users and non-IPT users who were attending health facilities of the Dessie city administration were considered as source populations. For IPT users, all adult PLHIV with IPT users who were attending health facilities of Dessie city administration were randomly selected at the time of data collection were considered as the study population. For non-IPT users,—all adult PLHIV with non-IPT users who were attending health facilities of Dessie city administration were randomly selected at the time of data collection were also considered as the study population. Non-IPT and IPT users of adult PLHIV who were attending health facilities of Dessie city administration at the time of data collection were included in the study.

### Sample size determination and sampling procedure

The sample size was calculated using a double population proportion formula with the proportion of TB among IPT users and non-IPT users in different studies, and then the larger sample size was taken. Based on the following assumptions: 95% level of confidence, 80% power, 1:1 ratio of IPT users to non-IPT users, and 10% non-response rate.

$$n= \left(\frac{\left(p1q1+p2q2\right)+ {\left(Z\beta +Z\alpha /2\right)}}{\left(p1-p2\right)2}\right)$$

Finally, the sample size with a larger number of IPT users and non-IPT users was selected, which was 148 with design effect of two due to multistage sampling technique applied and adding a non-response rate of 10%; then, the final sample size was 326 (163 IPT users and 163 non-users of IPT). The sample size was allocated to each selected health facility using proportionate allocation to the average monthly HIV-positive patient flow (IPT users and non-IPT users) from the registration book of ART follow up clinic in each health facility in an actual data collection period to make the sample representative. The study unit (HIV-positive patients) was selected using systematic sampling by determining the sampling interval (K). By calculating (K) as 3 for IPT users and 5 for non-IPT users, participants were selected at every third and fifth interval for IPT and non-IPT users in one month of the data collection period, respectively. When the data collector arrived at the place inside the room of ART clinic respondents who were taking care were considered the first HIV-positive patients to be interviewed.

### Variables of study

TB status was the outcome variable of the study. The independent variables were socio-demographic characteristics, clinical profile related factors, knowledge and behavioral characters related factors.

### Operational definition

#### ART adherence

For this research self- report ART medication were used. A good adherence was defined as not having missed even a single pill over the previous 4-day period on self-reporting^11,38^.

#### TB case diagnosis

Based on screening algorism of HIV positive patient, tuberculosis case finding for HIV positive was before and after start of ART by using a set of symptoms to identify tuberculosis and smear positive for AFB or Gene Xpert in pre-ART/ART register considered as TB positive.

#### Eligibility of IPT

All HIV patients are eligible for taking IPT when Patients have no history of active TB symptoms and no contraindications like: active hepatitis (acute or chronic) and symptoms of peripheral neuropathy^39^.

### Data collection tools and procedures

An interviewer-administered questionnaire was adapted by reviewing different literatures. The questionnaire included questions about the socio-demographic characteristics, clinical profile, and behavioral risk and knowledge-related characteristics of the study participants. Data were collected by 10 trained BSC nurses and supervised by 2 trained health officers. Face-to-face interviews supplemented by document review were conducted at the selected health facilities among HIV-positive patients who came to health facilities of ART clinics for refill or other service utilization.

The pretest was performed at Kombolcha health center by taking 10% (16 IPT and 16 non-IPT users) of the total sample size before the actual data collection to assess instrument simplicity, flow, and consistency of questions, and modifications were made accordingly. All questionnaires were checked for completeness every day by the principal investigator and supervisors. Training was given for data collectors and supervisors about the research objectives, data collection tools and procedures and interview techniques for one day. The principal investigator, together with two supervisors, supervised the technique of data collection and completeness of tools daily. The data were collected based on relevant guidelines and regulations which full fills the declaration of Helsinki principles in both IPT users and non-IPT users.

### Data processing and analysis

The data were checked for completeness and consistency in the field and coded, entered into Epi data version 3.1 software, and exported to Stata version 14 software for analysis. Descriptive statistics such as frequencies and percentages were calculated. Chi-square assumptions were first assessed for further analysis, and multicollinearity was checked using variance inflation factor (VIF). Variables with an average VIF above 5 were removed from the final analysis after checking those correlated variables with replacement independently. To identify the factors of the outcome variable, bivariable logistic regression was made, and those variables with P-value < 0.25 were entered into multivariable logistic regression analysis. The Hosmer–Lemeshow goodness of fit test for the final model was conducted for multiple logistic regressions to check the model fitness. Hence, the adjusted odds ratio with a 95% confidence level and the corresponding p-value was used to evaluate the association between independent variables and the outcome variable.

### Ethical consideration

Ethical clearance was obtained from the Ethical Review Committee of College of Medicine and Health Sciences, Wollo University. An official letter of cooperation was given to the administrative offices of the Dessie city, and written consent was obtained from the Dessie city administration, then provided to the respective health facilities. Prior to data collection the general purpose of the research was informed to each of the participants with a local language then written informed consent was obtained from each study subject. All information obtained from the individual was treated confidential and the anonymity of the participants was kept by using only coding without mentioning names during interview. They were informed that each participant has the right to take out from the study without constraint. For this purpose, a one-page consent letter was attached as a cover page of each questionnaire stating about the general objective of the study and issues of confidentiality which was discussed by the data collectors before proceeding with the interview.

## Supplementary Information

Supplementary Information.

## Data Availability

Datasets will not be shared to protect the participants’ confidentiality.

## Abbreviations

AIDS: Acquired immuno-deficiency syndrome
AOR: Adjusted odds ratio
ART: Antiretroviral therapy
CI: Confidence intervals
COR: Crude odds ratio
CPT: Cotrimoxazole preventive therapy
HIV: Human immuno-deficiency virus
IPT: Isoniazid preventive therapy
PLWHIV: People living with HIV
SPSS: Statistical Package for Social Sciences
TB: Tuberculosis
TST: Tuberculin skin test
VIF: Variance inflation factor
WHO: World health organization
