# Supplementary material for: Prevalence and associated factors of tuberculosis among isoniazid users and non-users of HIV patients in Dessie, Ethiopia
Source: Sci Rep. 2022 Aug 5;12:13500. doi: 10.1038/s41598-022-16437-3 (PMC9356010; doi:10.1038/s41598-022-16437-3)
Supplement: Supplementary file 1 — Supplementary Information. [file 41598_2022_16437_MOESM1_ESM.docx]

**Informed Consent Sheet**

My name is------------------------------- I am a data collector from temporarily working on behalf of principal investigator on the study with the objective of to asses the prevalence of tuberculosis and associated factors among PLWHIV IPT and Non-IPT Users in Dessie, City Northeast, Ethiopia , 2021 and I would like to ask you some question regarding the topic. The result of this study will help an input to improve quality of care related to PLWHIV and it is important to develop strategies that help to improve the prevention and control methods of TB among PLWHIV. Please assured that your name is not being recorded and any other identifying information will be kept confidential your participation is voluntary, and you have the right to not participate fully. You may stop the interview at any time. However, I hope that you will participate in this study since your views are important. May I begin the interview now? The interview will take 15-30 minutes.

Are you voluntary to participate? Yes _________ signature ______ continue to respond

No_____________ thank you

Date of interview (DD/MM/YY) -------------/------------------/2021

Name of interviewer…………………………………… Signature………………

Time started…………… Time ended………………

Name of supervisor…………………………....……………Signature……………….

Contact address cell phone 0935459310

**General information**

- Participant’s code number ___________________
- Woreda or Town administration_______________
- Kebele_________________

**Instruction**- circle the response for question with alternative and write for open ended question on the space provided

**Part 1: Socio-demographic characteristics**

| S.No | Question | Response |  |
| --- | --- | --- | --- |
| 1.1 | Sex | A. Male  B. Female |  |
| 1.2 | How old are you? (completed years) | ___________years |  |
| 1.3 | What is your place of residence? | 1. Rural 2. Urban |  |
| 1.4 | What is your level of education? | 1. Don’t write and read 2. Only read and write 3. Primary (1-4) 4. Primary (5-8) 5. Secondary (9-10) 6. Preparatory 7. Diploma or technical/vocational 8. Higher (bachelor degree and above) |  |
| 1.5 | What is your marital status? | 1. Single 2. Married   C. Widowed  D. Divorced  E. Separated |  |
| 1.6 | What is your current occupation status? | A. House wife  B. Merchant  C. Daily laborer  D. Governmental employee  E. Private/NGO employee  F. Others (specify__________) |  |
| 1.7 | Family size |  |  |
| 1.8 | Income |  |  |

**Part 2: Questions related to clinical and laboratory service**

| 2.1 | body mass index of participant? | …………………………kg/m2 |  | |  |  |
| --- | --- | --- | --- | --- | --- | --- |
| 2.2 | Current functional status participant? | 1. Working 2. Ambulatory 3. Bed reader |  | |  |  |
| 2.3 | Current WHO stage of participant? | 1. Stage 1 2. Stage 2 3. Stage 3 4. Stage 4 |  | |  |  |
| 2.4 | Have you ever experienced opportunistic infection? | 1. Yes 2. No |  | |  |  |
| 2.5 | If the above question is yes what type of OI? | ………………….. |  | |  |  |
| 2.6 | current CD4 count participant? ? | 1. <350 2. > 350 |  | |  |  |
|  | | | | |  |  |
|  | | | | |  |  |
| 2.7 | Did take cotrmoxzole perventive therapy? | A.Yes  B.No | |  |  |  |
|  |  |  | |  |  |  |
| 2.8 | Adherence for ART medication of participant? | A. Good  B .Poor | |  | |  |
| 2.9 | Do you screen for tuberculosis every visit for refill ART medication? | 1. Yes 2. No |  | |  |  |
| 2.10 | Did you diagnose having any Tuberculosis? | 1. Yes 2. No |  | |  |  |
| 2.11 | Question number 2.10 if yes how money times? | 1. 1 2. 2 3. 3 |  | |  |  |
| 2.12 | if Question number 2.10 yes , you treated tuberculosis? | A. Yes  B. No |  | |  |  |
| 2.13 | Question number 2.12 answer is yes what is final  outcome? | A. Cure  B. Failure  C. Default  D. Complete  E.On treatment |  | |  |  |
| 2.14 | Question number 2.13 answer is no why? |  |  | |  |  |
| 2.15 | Do you take isoniazid preventive therapy by health professional’s advice? | 1. Yes 2. No |  | |  |  |
| 2.16 | Do you take isoniazid preventive therapy ? | A. Yes  B. No |  | |  |  |
| 2.17 | Why you are not taking isoniazid preventive therapy? | A. Fear of side effect  B. Perversely TB treatment  C. Knowledge gap  D. Others……………… |  | |  |  |
|  | | | | |  |  |

**Part 3: Questions related to Knowledge on isoniazid preventive therapy and behaviors**

| 3.1 | Have you ever heard about isoniazid preventive therapy | A. Yes  B. No |  |
| --- | --- | --- | --- |
| 3.2 | Where did you first learn about isoniazid preventive therapy? (More than one answer is allowed) | A. News Media  B. Brochures, posters and other printed materials  C. Health workers  D. Family, friends, neighbors and colleagues |  |
| 3.3 | Have you heard any information about isoniazid preventive therapy | A. Yes  B. No |  |
| 3.4 | Ifyes for Q 3.3, from where get the information for the first time? | A. .Radio  B. .TV  C. Health professionals  D. .Relatives, colleagues and friends  E. .Others |  |
| 3.5 | Do you discuses about benefit of isoniazid preventive therapy with health worker? | A. Yes  B. No |  |
| 3.6 | Ifyes for Q 4.6 how money times? | A.. one time  B.. two time  C. three time  D .every visit |  |
| 3.7 | Did you think isoniazid preventive therapy to prevent tuberculosis | A. Yes  B. No |  |
| 3.8 | If the above question of 3.7 answer is No , why? | ………………………./ |  |
| 3.9 | Have you ever smoke? | A. Yes  B. No |  |
| 3.10 | If yes, how long you have been smoked? | ………………years |  |
| 3.11 | Question number 3.9 answer is yes have you heard any information smoking is not advice for client for anti-retro viral therapy | A. Yes  B. No |  |
| 3.12 | Have you ever drinking alcohol? | A. Yes  B. No |  |
| 3.13 | If yes, how long you have been drinking alcohol? | ………. Years |  |
| 3.14 | Question number 3.12 answer is yes have you heard any information alcohol is not advice for client for anti-retro viral therapy | A. Yes  B. No |  |

**Thank you**
